# ^1^H^N, 13^C, and^15^N resonance assignments of human calmodulin bound to a peptide derived from the STRA6 vitamin A transporter (CaMBP2)

**DOI:** 10.1007/s12104-019-09890-1

**Published:** 2019-03-14

**Authors:** Kristen M. Varney, Paul T. Wilder, Raquel Godoy-Ruiz, Filippo Mancia, David J. Weber

**Affiliations:** 1 Department of Biochemistry and Molecular Biology, Center for Biomolecular Therapeutics (CBT), University of Maryland School of Medicine, 108 N. Greene St., Baltimore, MD 21201 USA; 2 Department of Physiology and Cellular Biophysics, Columbia University, New York, NY 10032 USA

**Keywords:** Vitamin A, Retinoic acid, Calmodulin, STRA6

## Abstract

Vitamin A is a necessary nutrient for all mammals, and it is required for the transcription of many genes and vital for vision. While fasting, the vitamin A alcohol form (Retinol) from storage in the liver is mobilized and transported through the bloodstream while bound to retinol binding protein (RBP). Details of how exactly vitamin A is released from RBP and taken into the cells are still unclear. As part of the effort to elucidate the specifics of this process, single-particle cryo-electron microscopy structural studies of STRA6 (the RBP receptor 75-kDa transmembrane receptor protein) were recently reported by Chen et al. (Science, 10.1126/science.aad8266, 2016). Interestingly, STRA6 from zebrafish was shown to be a stable dimer and bound to calmodulin (CaM), forming a 180-kDa complex. The topology of the STRA6 complex includes 18 transmembrane helices (nine per protomer) and two long horizontal intramembrane helices interacting at the dimer core (Chen et al., in Science, 10.1126/science.aad8266, 2016). CaM was shown to interact with three regions of STRA6, termed CaMBP1, CaMBP2, and CaMBP3, with the most extensive interactions involving CaMBP2. To further our understanding of Ca^2+^-dependence of CaM-STRA6 complex formation, studies of the structure and dynamic properties of the CaMBP2–CaM complex were initiated. For this, the^1^H^N, 13^C, and^15^N backbone resonance assignments of the 148 amino acid Ca^2+^-bound calmodulin protein bound to the 27-residue CaMBP2 peptide derived from STRA6 were completed here using heteronuclear multidimensional NMR spectroscopy.

## Biological context

Vitamin A is required both for vision and to activate transcription via nuclear receptors such as the retinoic acid receptor (RAR) and the retinoid X receptor (RXR) (Chen et al. 2016; Palczewski 2012; Al Tanoury et al. 2013). Due to its involvement in diverse biological processes, disorders in vitamin A-dependent pathways result in disease states, including blindness and cancer (Shirakami et al. 2012; di Masi et al. 2015). Although a detailed mechanism for vitamin A transport is not fully elucidated, its cellular transport channel, STRA6, was proved necessary. STRA6 was shown to promote the release of retinol from the retinol binding protein (RBP), which transports and protects vitamin A in the extracellular space, and to transport retinol from extracellular to intracellular regions, where it is then received by the intracellular retinol binding protein (CRBP, notably CRBP1). Of note, this 75-kDa multipass transmembrane (TM) protein does not have sequence similarity to any other known transporter, channel, or receptor (Kawaguchi et al. 2008), and mutations in the human STRA6 gene have been linked to Matthew–Wood syndrome (MWS), which presents with ocular defects ranging from mild microphthalmia to anophthalmia, as well as with an array of other developmental abnormalities including cardiac and pulmonary defects and cognitive deficits (Chassaing et al. 2009). Recently, Chen et al. solved the 3.9 Å cryo-electron microscopy structure of the STRA6 dimer, which was found to be in tight and physiological association with Ca^2+^-bound calmodulin (CaM) (Chen et al. 2016). Interestingly, Ca^2+^-CaM adopts a novel conformation when bound to STRA6, so further studies on this complex will prove vital in determining the role of CaM and Ca^2+^ in STRA6 function. Specifically, NMR dynamics-based work on this complex is aimed at investigating structure/function relationships of the Ca^2+^–CaM–STRA6 complex, which may be important for further delineation of the mechanism of action of STRA6-dependent vitamin A transport in mammals.

## Methods and experiments

### Sample preparation

The expression vector for hCaM was generously supplied by Dr. L. Mario Amzel at Johns Hopkins University in a pET24 plasmid without any affinity tag, which was transformed and expressed in *E. coli* strain BL21 (DE3). A single colony of this bacteria was used next to inoculate MOPS minimal media containing just^15^NH_4_Cl as the sole nitrogen source for the expression of ^15^N-labeled hCaM (148 total amino acid construct). For [^13^C,^15^N]-doubly labeled hCaM preparations, 2.5 g/L^13^C_6_-glucose and 1.0 g/L^15^NH_4_Cl were used as the sole carbon and nitrogen sources, respectively. Expression of either^15^N-labeled or [^13^C,^15^N]-labeled hCaM proteins was induced with 0.5 mM isopropyl-β-d-thiogalactopyranoside (IPTG) addition at 25 °C and then grown overnight. Bacterial cultures were pelleted at 4 °C by centrifugation and resuspended in lysis buffer (50 mM Tris, pH 7.5, 1 mM DTT, 5 mM EDTA, 1 mM PMSF) supplemented with 0.1 mg/mL of lysozyme, 10 units of DNAse, 10 mM MgCl_2_ and 5 mM CaCl_2_. The cells were lysed further by sonication and cell debris was separated by centrifugation at 15,000 rpm for 45 min at 4 °C. The supernatant was collected, kept cool on ice, and 10% streptomycin sulfate solution was added slowly for 30 min with stirring. Precipitated DNA was removed by centrifugation at 15,000 rpm for 45 min. The soluble fraction was dialyzed overnight against 4L of Buffer A [50 mM Tris, pH 7.5, 10 mM β-mercaptoethanol (β-ME)] and applied to a HiPrep DEAE FF 16/10 Sepharose column (GE Healthcare, #28936541) previously equilibrated with buffer A. hCaM fractions were eluted from the DEAE column using a linear gradient of 10–35% Buffer B (50 mM Tris, pH 7.5, 10 mM β-ME, 1 M NaCl), pooled and dialyzed against Buffer C (10 mM Tris, pH 7.5, 500 mM NaCl, 10 mM CaCl_2_, and 0.25 mM DTT). The dialyzed protein was then applied to a HiPrep Phenyl Sepharose FF (High Sub) 16/10 column (GE Healthcare, #28936545) and eluted from the column in a single step using 100% Buffer D (10 mM Tris, pH 7.5, 500 mM NaCl, 10 mM EDTA, and 0.25 mM DTT). As a final purification step, the pooled Phenyl Sepharose fractions were concentrated and injected onto a Superdex 200 16/600 (S200-PG) column (GE Healthcare, #28989335) previously equilibrated with Buffer E (20 mM HEPES, pH 7.4, 50 mM NaCl, 0.5 mM TCEP and 0.02% NaN_3_). Fractions showing pure hCaM were concentrated to ≈ 1–2 mM and desalted via a 10 mL Sephadex G-25 column (GE Healthcare, 17-0033-01) equilibrated with chelexed-buffer E. The protein was then dialyzed, concentrated, aliquoted, and stored at − 80 °C. CaM concentrations were determined by Bradford Assays in which CaM samples of known concentration, via amino acid analyses, were used as the standard. Purity was demonstrated by SDS-PAGE (> 99%). A typical NMR sample contained 0.2 mM hCaM in 20 mM HEPES, pH 7.4, 50 mM NaCl, 10 mM CaCl_2_, 5 mM MgCl_2_, 0.5 mM TCEP, 10% D_2_O and 0.43 mM CaMBP2 peptide. The CaMBP2 peptide consists of amino acid residues 554–571 from STRA6 (554-VSNAKRARAHWQLLYTLVNNPSLVGSR-571) as synthesized using solid-state peptide synthesis, and its purity was determined to be > 95% by high pressure liquid chromatography and mass spectrometry (Biosynthesis Inc., Lewisville, TX or GenScript, Piscataway, NJ). Since this is an internal peptide from STRA6, the C-terminus was amidated, and its N-terminus was acetylated to neutralize the charged termini. The concentration of the stock solutions of unlabeled peptides was determined by quantitative amino acid analysis (Biosynthesis Inc., Lewisville, TX).

### NMR experiments

**Fig. 1:**
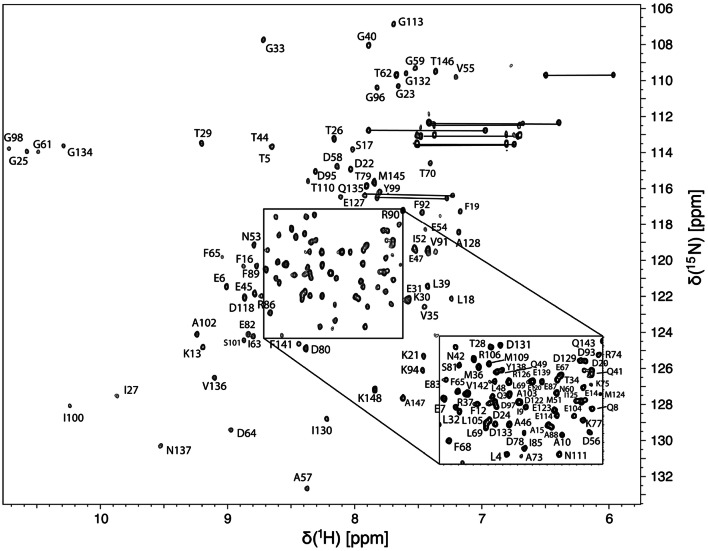
2D [^1^H-^15^N]-fHSQC of Ca^2+^-calmodulin bound to CaMBP2 recorded on a Bruker Avance III 950 MHz spectrometer at pH 7.4 and 298 K. Backbone amide^15^N–^1^H correlations are labeled with the single-letter amino acid code and residue number of the mature native protein

**Fig. 2:**
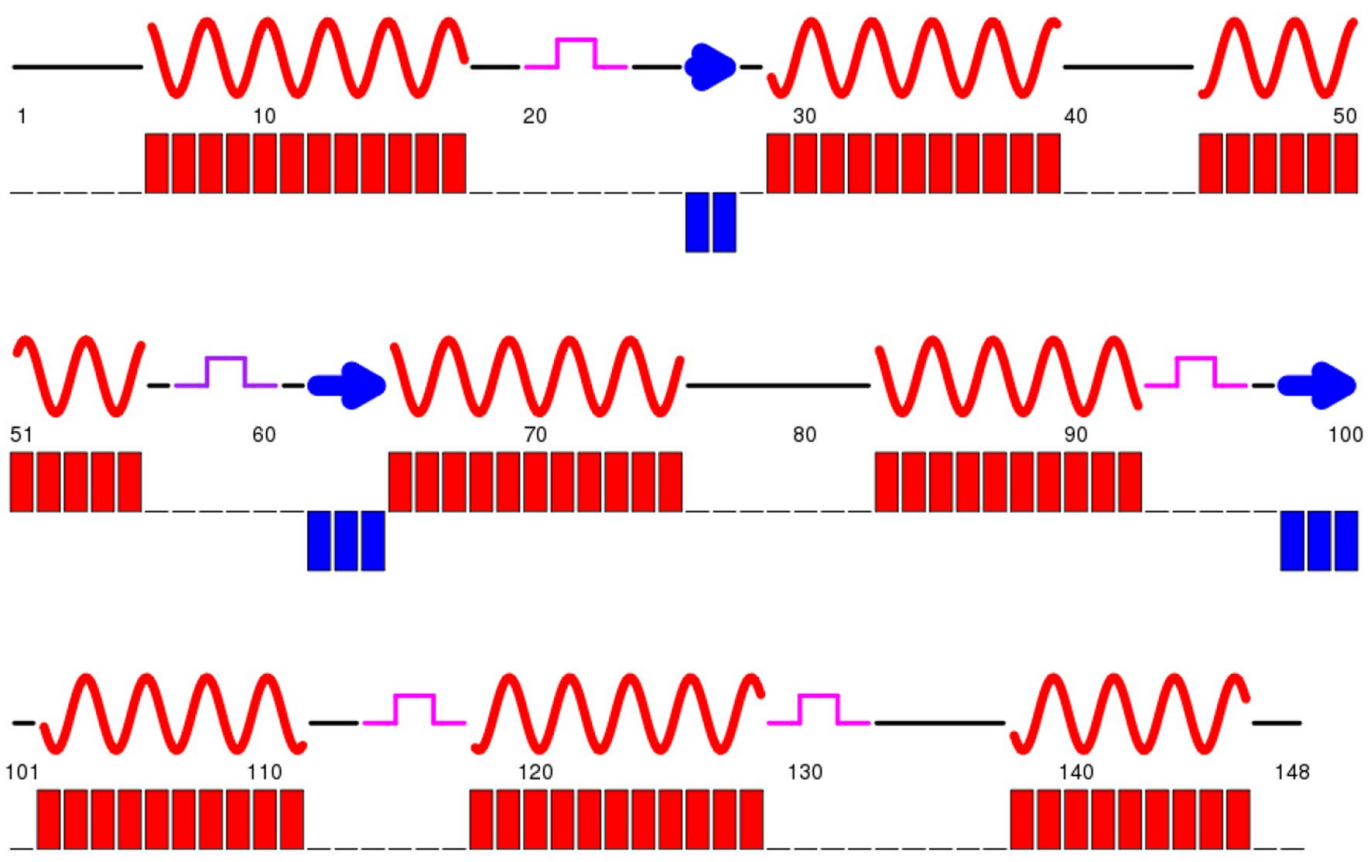
Characterization of Ca^2+^-calmodulin bound to CaMBP2 peptide based on NMR chemical shifts. Raw chemical shift deviations of C*α* and Cβ carbons (Δδ(C*α*) − Δδ(Cβ)) with respect to corresponding random coil values are plotted against residue number. Positive and negative values indicate*α*-helix and β-strand character, respectively. Probability of secondary structure formation as predicted by CSI3.0, with *α*-helices represented by red curves and β-strands by blue arrows

All NMR experiments were acquired at 298 K on a Bruker Avance III 950 MHz spectrometer equipped with a *z*-gradient cryogenic probe. A 2D [^1^H–^15^N]-fHSQC, shown in Fig. 1, was used as the root spectrum to assign backbone resonances via pairwise comparison of inter- and intra-residue ^13^C*α*, ^13^Cβ and^13^Cʹ chemical shifts. Triple resonance HNCACB, CACB(CO)NH, HNCA, HN(CO)CA, HNCO, and HN(CA)CO experiments were collected on [^15^N,^13^C]-labeled CaM–CaMBP2 samples (0.2 mM hCaM complexed with 0.43 mM CaMBP2 peptide in 20 mM HEPES, pH 7.4, 50 mM NaCl, 10 mM CaCl_2_, 5 mM MgCl_2_, 0.5 mM TCEP, 10% D_2_O) at 25 °C. CSI3.0 was used to determine secondary structure probabilities based on experimentally derived H^N^, N, C*α*, Cβ and Cʹ chemical shifts (Fig. 2). NMR data were processed with NMRPipe (Delaglio et al. 1995) and analyzed with CcpNmr Analysis (Vranken et al. 2005). All proton chemical shifts were referenced to external trimethylsilyl propanoic acid (TSP) at 25 °C (0.00 ppm) with respect to residual H_2_O (4.698 ppm).^1^H–^15^N and^1^H–^13^C chemical shifts were indirectly referenced using zero-point frequency ratios of 0.101329118 and 0.251449530, respectively.

### Assignments and data deposition

Backbone assignments were obtained for the Ca^2+^-calmodulin protein bound to the STRA6 CaMBP2 peptide in order to serve as a starting point for studies to elucidate the backbone dynamical behavior of Ca^2+^-calmodulin in this complex. The well-dispersed 2D [^1^H–^15^N]-fHSQC spectrum of the 148-residue calcium-binding protein CaM is shown in Fig. 1 when it is bound to unlabeled CaMBP2 and Ca^2+^. Under conditions used in this experiment, 100% (132/132) of the observable^1^H–^15^N correlations were assigned unambiguously with the remaining 16 residues either not observed due to exchange broadening (A1, D2, M71, M72, M76, E84, H107, V108, L116, T117, E119, V121, E140, M144) or absent because they are proline residues (P43, P66). Further, 96%, 92% and 96% of all observable C*α*, Cβ and Cʹ chemical shifts, respectively were assigned unambiguously. The chemical shift assignments from these experiments were deposited in the BioMagResBank (http://www.bmrb.wisc.edu) under accession number 27782. The chemical shift assignments determined here were used to generate a chemical shift index and map secondary structure. As shown in Fig. 2, the predicted secondary structure of this novel CaM fold is predominantly helical and consistent with that of the CaM–CaMBP2 crystal structure and the cryoEM structure of the full-length CaM–STRA6 complex (Chen et al. 2016). Specifically, it is comprised of eight alpha helices (E6-S17; T28-L39; E45-V55; F65-K75; E83-F92; A102-F111; D118-A128 and Y138-T146) and two beta strands (T62-D64 and G98-I100).
