# Correction to: ^1^H^N^, ^13^C, and ^15^N resonance assignments of human calmodulin bound to a peptide derived from the STRA6 vitamin A transporter (CaMBP2)

**DOI:** 10.1007/s12104-020-09942-x

**Published:** 2020-04-04

**Authors:** Kristen M. Varney, Paul T. Wilder, Raquel Godoy-Ruiz, Filippo Mancia, David J. Weber

**Affiliations:** 1 Department of Biochemistry and Molecular Biology, Center for Biomolecular Therapeutics (CBT), University of Maryland School of Medicine, 108 N. Greene St., Baltimore, MD 21201 USA; 2 Department of Physiology and Cellular Biophysics, Columbia University, New York, NY 10032 USA

## Correction to: Biomolecular NMR Assignments (2019) 13:275–278 10.1007/s12104-019-09890-1

The article ^1^H^N^, ^13^C, and ^15^N resonance assignments of human calmodulin bound to a peptide derived from the STRA6 vitamin A transporter (CaMBP2), written by Kristen M. Varney, Paul T. Wilder, Raquel Godoy-Ruiz, Filippo Mancia and David J. Weber, was originally published Online First without Open Access. After publication in volume 13, issue 2, page [275–278] the author decided to opt for Open Choice and to make the article an Open Access publication. Therefore, the copyright of the article has been changed to © The Author(s) 2020 and the article is forthwith distributed under the terms of the Creative Commons Attribution 4.0 International License (https://creativecommons.org/licenses/by/4.0/), which permits use, duplication, adaptation, distribution and reproduction in any medium or format, as long as you give appropriate credit to the original author(s) and the source, provide a link to the Creative Commons license, and indicate if changes were made. The original article has been corrected.

